# Neural changes to transcranial alternating current stimulation in the gamma range over the left frontoparietal network: A preliminary eLORETA EEG study

**DOI:** 10.3934/Neuroscience.2026012

**Published:** 2026-05-15

**Authors:** Tien-Wen Lee, Gerald Tramontano

**Affiliations:** The NeuroCognitive Institute (NCI) Clinical Research Foundation, Mt Arlington, NJ 07856, USA

**Keywords:** electroencephalography (EEG), exact Low-resolution electromagnetic tomography (eLORETA), frontoparietal network, transcranial alternating current stimulation (tACS), transcranial electrical stimulation (tES)

## Abstract

Applying transcranial alternating current stimulation (tACS) at the gamma range to the frontal and parietal regions can improve cognitive dysfunctions. This study aimed to explore the neural changes following tACS. Electroencephalography (EEG) recordings were obtained from a cohort of 34 participants with various cognitive impairments before and after 11 sessions of 40 Hz tACS treatment. Alternating currents at 2.0 mA were administered to the electrode positions F3 and P3 for 25 min of each session, following the 10–20 EEG convention. Using eLORETA, scalp-recorded signals were reconstructed into cortical current source density (CSD). We then assessed the differences in power and connectivity strength across multiple spectra. We observed a consistent trend of decreased CSD at the stimulating sites across different spectra, most prominent at beta and gamma bands (*P* < 0.01). On the contrary, the right hemisphere showed a trend of increased CSD, which was likely mediated by inter-hemispheric rivalry. In addition, the connectivity strength between the left frontal and parietal regions increased significantly (*P* = 0.017). Application of tACS would desynchronize regional oscillation and enhance inter-regional crosstalk. The pattern of neural changes was concordant with our previous tACS reports (5 Hz), suggesting common neural mechanisms driving the neurophysiological effects of tACS.

## 1. Introduction

Neural oscillations across diverse frequency bands are intrinsic to brain dynamics and critically support various domains of cognition [1]–[3]. In terms of working memory, executive functions have been associated with theta phase coupling between frontal and posterior brain regions [4]. In addition, frontal midline theta EEG activity may control activity in the parietal cortex associated with memory maintenance [5]. Alpha band is linked to semantic memory and attentional processes [2], while beta oscillations are implicated in sensorimotor functions, top-down processing, and preservation of the current brain state [6]. Gamma-frequency activity is thought to support the coordination of distributed brain networks and has been implicated in cognitive domains such as attention, memory maintenance, and perceptual binding [7]–[9]. Moreover, aberrant gamma activity has been associated with cognitive dysfunction across a range of neurocognitive disorders [10]–[14]. The emergence of transcranial alternating current stimulation (tACS) now allows researchers to artificially modulate neural rhythms and assess their causal role in cognition by entraining brain activity at specific frequencies [15]–[17].

Although transcranial electrical stimulation (tES) has been widely applied as a tool for basic research [18], studies have supported tACS as a treatment option for neuropsychiatric conditions [19]–[21]. Gamma tACS has been shown to improve the performance of working memory, event memory, visuomotor learning, and fluid intelligence [22]–[26]. Previous reports suggested that tACS at 40 Hz was promising in the prevention and treatment of Alzheimer's disease [27],[28] and various psychiatric conditions [21],[29],[30]. Our recent clinical study delivered 40 Hz tACS to cognitively impaired populations and supported its efficacy and potential in clinical practice [31]. Together, applying gamma tACS to the frontoparietal network may benefit heterogeneous populations across a wide age range, including healthy individuals as well as those with attention-deficit/hyperactivity disorder, learning disabilities, Alzheimer's disease, and various other neuropsychiatric conditions [23]–[25],[27],[28],[31].

The broad utility of tACS over frontoparietal regions appears to enhance cognition irrespective of specific diagnoses. The observed benefits are more likely attributable to compensatory adaptations driven by enhanced frontoparietal network functioning, rather than the direct remediation of underlying pathologies. However, the neural mechanisms supporting these effects remain unclear. It is observed that tACS may change the firing rate and spike timing of neuronal activities in a spectra-specific manner [32]–[35]. It was thus inferred that applying a particular frequency of tACS can *synchronize* the neural oscillations to match the frequency of the electrical stimulation, framed as *entrainment theory* [36],[37]. The evidence of tACS-induced entrainment in the human brain dynamics, however, has been controversial [20],[38]–[41]. Our previous EEG research of 5 Hz tACS over the right hemisphere has provided novel insight into this pivotal issue [42],[43]. By comparing the EEG collected before and after 25 min tACS at 5-Hz (in theta range), we observed spectrum-specific and unspecific alterations, summarized below. First, tACS at 5 Hz desynchronized regional theta power and enhanced contralateral theta power. The latter was likely mediated by the neural mechanism of inter-hemispheric rivalry [44]–[46]. The areas undergoing modulation were widespread, as opposed to the more focal transcranial magnetic stimulation. Second, tACS imposed cross-spectral influence on power, well beyond the “entrained” frequency—spectral-unspecific. Last, inter-regional connectivity (phase synchronization) increased in a spectrum-specific way, which only occurred at the theta range.

Continuing from our previous work, this research explored the neural consequences of 40 Hz gamma tACS applied over the left frontoparietal network across 12 treatment sessions, aiming to enhance cognitive capability. Both regional power and inter-regional connectivity profiles were examined. In line with the brain-based approach in our previous report (in contrast to scalp-based analog), we utilized exact low-resolution brain electromagnetic tomography (eLORETA) to map EEG data from the scalp onto the gray matter voxels of a standardized brain template, i.e., current source density (CSD) [47],[48]. The power changes of CSD following treatment were examined statistically. The connections between the frontal and parietal areas, characterized by different frequency spectra, were assessed using lagged coherence, which is a linear measure, and phase synchronization, a nonlinear indicator of functional connectivity [49]. Following our previous EEG research exploring the neural influence of theta-range tACS, we hypothesized that gamma tACS would analogously produce the following effects: (1) the left and right frontoparietal networks would show opposite trends of power changes, with tACS exerting an unfavorable influence on the underlying neural synchronization; (2) the power modulation would not be limited to the gamma range but would extend to lower frequency ranges (i.e., cross-spectral effects); and (3) functional connectivity strength would increase between left frontal and parietal regions.

## 2. Materials and methods

### 2.1. Participants and neuropsychological assessment

#### 2.1.1. Participant characteristics

This study focused on patients exhibiting cognitive deficits who underwent 40 Hz tACS treatment applied to the left frontal and parietal regions. To be included in this study, patients needed to have a diagnosis characterized by cognitive deficits [e.g., mild cognitive impairment (MCI), learning disability, or attention-deficit hyperactivity disorder (ADHD)] or a chief complaint of cognitive decline. To provide complementary behavioral context to the electrophysiological findings, selected tasks from the D-KEFS were included [50]. The behavioral battery included Trails A and B, Letter Fluency, and Design Fluency tests. In Trails A, participants were asked to sequentially connect numbered circles as quickly and accurately as possible, while Trails B required alternation between numbers and letters, assessing both processing speed and cognitive flexibility, with results reported in seconds. In the Letter Fluency task, participants were asked to generate as many words as possible beginning with a specified letter within a fixed time, evaluating verbal fluency. The Design Fluency task required participants to produce unique non-verbal designs under time constraints, assessing non-verbal executive function. For both fluency tasks, results were reported as the number of correctly produced items. Detailed procedures for these tasks are provided in the original battery. Individuals with epilepsy, skull defects, intracranial electrodes, brain lesions, vascular clips or shunts in the brain, cardiac pacemakers, or other implanted biomedical devices, as well as those who were pregnant or lactating, were deemed ineligible for tES in accordance with safety guidelines [51],[52]. Participants were instructed to maintain their current treatments, including medications, without any dosage adjustments during the tES treatment. The primary focus of this research was the changes in EEG following tES. We identified 34 cognitively impaired patients who received full tACS treatment and underwent pre- and post-treatment EEG.

#### 2.1.2. Ethics approval of research

We reviewed data collected from our clinics between 2018 and 2022, following prior approval of the research protocol by the private review board Pearl IRB, with approval ID 2023–0133. Written informed consent was obtained from all clinical patients. For participants under 18 years of age, the consent was provided by their parents, legal guardians, or next of kin, in accordance with ethical guidelines requiring appropriate consent for minors.

### 2.2. Administration of tACS

We employed an Investigation Exempt Device (IDE), the Starstim-8 neuromodulation device, developed by Neuroelectrics, Inc. (Barcelona, Spain), to attenuate cognitive deficits arising from different causes. The device utilizes electrodes connected through wires to a rechargeable battery, delivering electric currents directly to the scalp and brain. Prior to attaching the electrodes, the scalp underwent a gentle cleansing with skin preparation gel. Subsequently, conductive gel was applied to facilitate proper electrode-to-scalp contact. These steps were essential to maintain optimal electrode contact and ensure that the impedance level remained below 5 kΩ [53].

**Figure 1.**
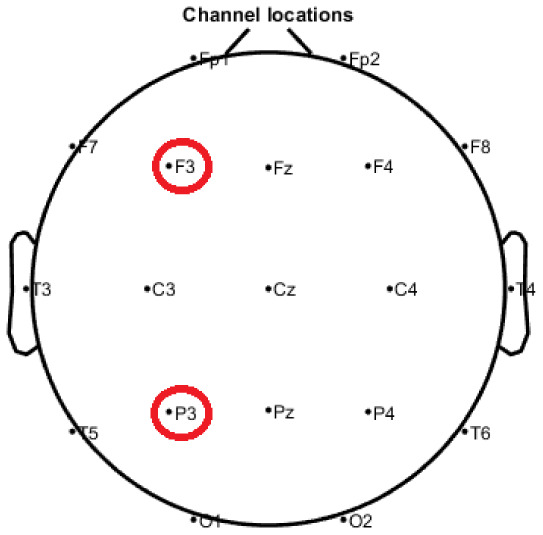
Illustration of the F3 and P3 positions on the scalp.

Stimulation was delivered using circular Pistim electrodes with a contact area of π cm² (3.14 cm²), positioned in a neoprene cap. The montage of electrodes covered the left lateral side of the head at F3 and P3 positions in terms of the 10–20 EEG convention. The peak current intensity was 2.0 mA, with sinewave currents oscillating at 40 Hz and alternating between electrodes F3 and P3 (see Figure 1). To familiarize patients with tES, we implemented a gradual dose escalation strategy during the initial three sessions. This strategy involved the following current levels: 1.0 mA for the first session, 1.5 mA for the second session, and 2.0 mA for the third session. Each stimulation session had a duration of 25 min, commencing with a 1-min ramp-up phase and concluding with a 30-s ramp-down phase to minimize skin irritation. The 12 treatment sessions were completed in 3 weeks. The second EEG was recorded immediately before the final treatment session to assess the cumulative effect of the entire protocol. This timing was chosen to minimize potential short-term session effects associated with the last stimulation, such that the measured changes primarily reflect the cumulative influence of the preceding 11 sessions. Importantly, it was not acquired after the last session to avoid confounding session-specific effects with the longer-term effects of the full protocol. The interval between the eleventh treatment and the second EEG recording was 1–3 days.

### 2.3. EEG recording and data preprocessing

Before the initial treatment and right before the last neuromodulation session, we employed the Brainmaster device (Discovery 24, with bandwidth 0–80 Hz, 19 channels; https://brainmaster.com/) to collect 10-min EEG data with eyes open at a sampling rate of 256 samples per second, utilizing a linked-ear reference. EEG recording involved the following steps: Participants were seated comfortably, their scalp was cleaned, and a customized electrode cap was aligned according to the 10-20 system. Conductive gel was applied, and impedance was checked to ensure it remained below 5 Ω. Signal quality was assured and continuously monitored throughout the recording to ensure clarity and participant compliance. We utilized the EEGLAB software to edit the EEG traces [54]. Data preprocessing included band-pass filtering (1–50 Hz), automatic artifact removal [55],[56], and manual elimination of the remaining noisy portions, including those linked to blinks, flutter, eye movements, line movement, and sweat artifacts, among others. The above-mentioned processing was conducted by a single rater (TW Lee). After eliminating artifacts, the cleaned EEG data were divided into 2-s epochs and then imported into eLORETA for further analysis.

### 2.4. eLORETA and power analyses

The eLORETA method was employed to transform scalp EEG signals into neural electrical activities of 6239 gray matter voxels of an MNI template [47],[48]. Unlike the conventional parametric approach of source localization, eLORETA utilizes a linear imaging method with adaptive data-dependent weights to account for measurement and biological noise. It accurately computes arbitrary point-test sources with precise, error-free localization. eLORETA, based on linearity and superposition principles, is well-suited for mapping distributed electric sources (CSD) in the brain cortex, albeit with limited spatial resolution. The power spectra of delta (1–4 Hz), theta (4–8 Hz), alpha (8–12 Hz), beta (12–30 Hz), and gamma (30–50 Hz) for each voxel were derived [57].

We used paired t-tests and a nonparametric statistical method (SnPM) with 5000 randomizations to compare cortical CSD powers before and after treatment [58],[59]. The max-statistics and corrected *P*-values for multiple testing can thus be derived. We employed the exceedance proportion test in eLORETA software to assess the significance of activity based on its spatial extent, identifying clusters of supra-threshold voxels. The null hypothesis was that there were no differences in CSD powers after tES treatment, with a two-tailed significance level of *P* < 0.05. A more detailed explanation of the principles behind eLORETA and the nonparametric exceedance proportion test can be obtained in our recent report and source papers [43],[47],[59].

### 2.5. Connectivity analysis

We used lagged (general) coherence and phase synchronization to represent linear and nonlinear functional connectivity strength, respectively [49]. Both were computed from the CSD time series of the selected voxel. The equations for phase synchronization are similar to those for coherence, except for a pre-normalization step that accounts for power influence, making it a measure of nonlinear connectivity unaffected by amplitude relationships. Total coherence comprises both lagged and instantaneous dependence. However, because the instantaneous part is influenced by non-physiological factors like volume conduction and low spatial resolution, it was omitted in this report [49]. For the same reason, we only focused on the lagged component of phase synchronization.

The average connectivity strengths were calculated between two coordinates, which were the center of Brodmann area (BA) 8/9 [frontal; (−30, 30, 40)] and BA 39/40 [parietal; (−45, −50, 40)], by averaging the values of all functional connectivity indices across the five defined frequency bands, as used in the power spectral analyses. Paired t-tests were used to examine the connectivity changes before and after the tACS treatment course.

## 3. Results

The age of the 34 patients ranged from 10.9 to 84.0 years, with a mean ± SD of 60.0 ± 24.7 years. Among them, there were 15 males and 19 females. All selected patients tolerated the maximum tES current at 2.0 mA. Their diagnoses included MCI (n = 12), ADHD (10), mild to moderate intellectual disability (4), learning disability (6), and traumatic brain injury (2). Only mild tACS-related side effects, such as tingling or itchiness, were observed, as in our previous report [21],[31]. Changes in test scores were observed for Trail A, Trail B, and Letter Fluency, but not for Design Fluency (see Table 1).

**Table 1.** Comparative results of selected neuropsychological tests pre- and post-treatment (post–pre differences).

	Pre-Tx	Post-Tx	t-scores	*P*-values
Trail A (sec)	40.8 (19.0)	33.5 (14.0)	−2.85	0.0079
Trail B (sec)	116.9 (60.3)	97.3 (42.9)	−3.48	0.0016
Letter Fluency	28.0 (10.7)	31.3 (10.9)	3.20	0.0033
Design Fluency	24.2 (8.6)	26.9 (9.2)	2.04	0.0510

Note: Tx: treatment; subject number = 30.

### 3.1. Power analysis and statistical comparisons

**Figure 2.**
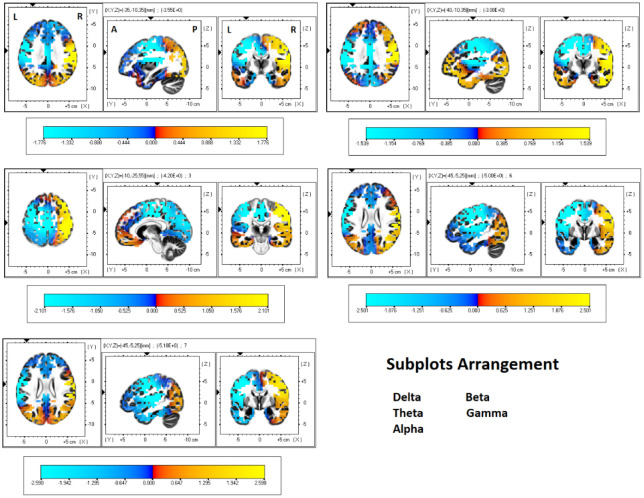
Changes in CSD powers (post-treatment minus pre-treatment) represented at five defined spectra (delta, theta, alpha, beta, and gamma). Warm colors indicate an increase, while cool colors indicate a decrease. The axial (left), sagittal (middle), and coronal (right) sections are centered around the highest statistics. A color bar is included at the end of each sub-graph, scaled to 50% of the maximum of t-scores to provide a clearer illustration of the trend in CSD changes for each frequency band.

**Figure 3.**
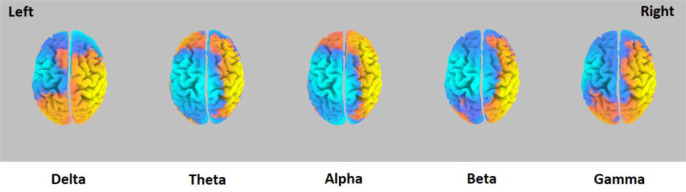
Top views of the CSD differences following tACS treatment, highlighting a consistent trend of power changes across different spectra. Due to the difficulty of incorporating five different color bars with varying scales in this figure, the color bars are referred to in Figure 2.

The neural responses to 40Hz tACS exhibited a consistent pattern across different spectra: (1) at the left frontoparietal region, the same side of tES, there was a significant reduction in CSD powers at gamma and beta frequencies. Both the peak t-values and the spatial extents surpassed the statistical thresholds, with *P*-values at the level of 0.005. Notably, the trend propagated to lower frequency bands. (2) At the right frontoparietal region, contralateral side of tES, there was a significant enhancement of CSD powers. The peak t-values did not survive the statistical challenge, but the spatial extent remained significant. Again, the observed trend extended to lower frequency bands. The results of power analyses are summarized in Table 2 and illustrated in Figures 2 and 3.

**Table 2.** Summary of neural changes to neuromodulation treatment, with the points with the highest t-values reported.

	Delta	Theta	Alpha	Beta		Gamma	
Regional peak t value	−3.55	−3.08	−4.20	−5.00*	3.65^†^	−5.10*	2.91^†^
Extreme *P*-value (2-tailed)	0.249	0.549	0.055	0.005	0.255	0.005	0.705
Coordinate	−35,−10,35	−40,−10,35	−10,−25,55	−45,−5,25	40,−40,65	−45,−5,25	65,−25,40
Brain region	precentral g.	precentral g.	mfrontal g.	precentral g.	postcentral g.	precentral g.	precentral g.
BA at peak coordinate	6	6	6	6	2	6	1
Spatial extent (voxels)	37	21	230	444	219	226	112
BA: spatial extent	6,13,24	6	3–6,24,31	4,6–9,13,24,43–45	1–5,40	1–6,9,13,40–44	1–4,40

Note: The coordinates are based on the Montreal Neurological Institute (MNI) template and follow the RAS convention (positive: right, anterior, and superior). The extreme *P*-values are multi-comparison corrected, with the threshold t = 4.42 and *P* = 0.05. The spatial extent derived from the exceedance proportion test is thresholded at t = 2.60 with *P* = 0.05. BA: Brodmann area; mfrontal: medial frontal; g.: gyrus. * Both peak and extent *P*-values < 0.05, ^†^ extent *P*-value < 0.05.

### 3.2. Connectivity analysis and statistical comparisons

**Figure 4.**
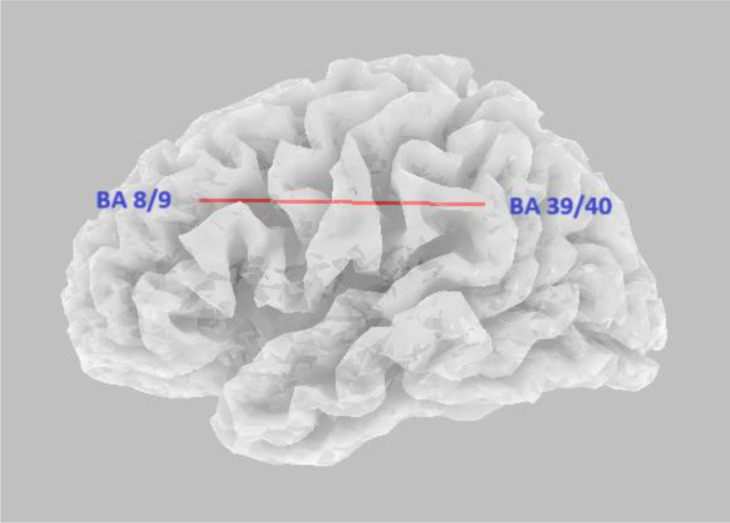
Lagged phase synchronization was increased in the frontoparietal network. Centers of Brodmann areas serving as neural nodes for connectivity analyses are labeled.

Our hypothesis that the connectivity strengths were increased after the 40 Hz tACS over the frontoparietal network was supported. The enhancement was statistically significant for phase synchronization, not coherence. The connectivity strengths and the statistics are summarized in Table 3, and the significant results are illustrated in Figure 4. Supplementary analyses showed that the connectivity changes were only present in the gamma range, not in other spectra (spectrum-specific; data not shown).

Given the positive findings in lagged phase synchronization, we examined the correlations between the magnitude of connectivity changes and the score changes on the four selected tasks. The correlation coefficients for Trail A, Trail B, Letter Fluency, and Design Fluency were 0.20, 0.10, 0.33, and −0.07, with corresponding *P*-values of 0.258, 0.587, 0.0564, and 0.710, respectively.

**Table 3.** Lagged general coherence and phase synchronization were computed between the central coordinates of BA 8/9 and BA 39/40 and were compared between post-treatment and baseline conditions.

	Mean (SD)		t score	*P*-value
Lagged general coherence				
	Post-tACS	Baseline		
BA8/9 and BA39/40	0.0027 (0.002)	0.0022 (0.002)	1.05	0.302
Lagged phase synchronization				
	Post-tACS	Baseline		
BA8/9 and BA39/40	0.0024 (0.002)	0.0014 (0.001)	2.50	0.018*

Note: * *P*-value < 0.05. BA: Brodmann area.

## 4. Discussion

Neural oscillation is a fundamental characteristic of living organisms [60], endorsing various cognitive functions and underlying abnormalities in neurocognitive disorders [27],[57]. In addition to offering a research gateway to modulate brain rhythms and cognitive processes, tACS at the gamma range to the frontoparietal network can improve clinical cognitive dysfunctions across diverse diagnoses and age ranges [17],[23]–[25],[31],[37]. By adopting a self-controlled design (within-subject control), the authors utilized EEG to investigate neural alterations subsequent to 11 treatment sessions of 2 mA tACS at 40 Hz over left frontal and parietal regions, which aimed to ameliorate cognitive deficits resulting from diverse diagnoses. Through the comparisons of post-treatment minus baseline neural indices, our three hypotheses regarding power and connectivity were supported, aligning closely with the findings from our previous 5 Hz tACS and EEG studies that employed a similar experimental design and analytic strategy [42],[43]. As both tES and eLORETA are low-resolution tools, we discussed the results on a lobar or hemispheric scale, instead of focusing on the peak coordinates, which was the typical approach in previous neuroimaging studies. The present design enables us to examine within-subject EEG changes induced by gamma tACS. Whether the magnitude of these effects is influenced by demographic variables (e.g., age or gender) or by different clinical diagnoses remains an important question but is beyond the scope of this preliminary report.

### 4.1. Power spectra changes associated with tACS treatment

We noticed reduced CSD powers on the same side of alternating current application and increased powers on the opposite side of the brain. This pattern was most pronounced in the gamma and beta frequency ranges, and a similar trend was also observed in the lower frequency bands, from delta to alpha. The findings offer two important insights. First, artificial injection of alternating currents into the brain interfered with, rather than synchronized, underlying neural oscillation across spectra. Although increased neural oscillation to tACS had been inferred from animal studies and entrainment theory (mainly from real-time recordings) [32]–[37], recent human research has not consistently validated the prediction but has instead suggested alternative neural mechanisms in the offline conditions, such as plasticity. For example, application of alpha tACS targeting the dorsolateral prefrontal cortex decreased the alpha power [20]. Delivering tACS at 6 and 10 Hz over the occipito-parietal area impaired performance in the detection task, indicating interference rather than facilitation of the underlying neural processing [40]. Lafton et al. conducted intracranial recordings and found no evidence of sleep rhythm entrainment induced by tACS [41]. Given that neural oscillations are built upon intricate molecular, cellular, and intercellular infrastructures and conform to electrophysiological constraints [60]–[62], it is reasonable to assume that injecting artificial alternating currents will force the neural dynamics away from their balanced optimal state, being unfavorable for neural synchronization.

Interestingly, the contralateral side of the administered tACS presented with enhanced CSD powers across spectra, again most prominent for beta and gamma, and the trend persisted to the lower frequency range. The bi-hemispheric reverse pattern of power response to tACS is a frequently observed phenomenon in the field of tES research. The most probable neural mechanism to mediate this is through inter-hemispheric rivalry [44]–[46]. The induced transient reduction in neural activity in one region will disrupt the dynamic equilibrium, and the ensuing decreased inhibitory output will enhance the neural activity in the homologous area of the contralateral hemisphere. The inverse trend of power changes also contributes to keeping the brain's energy expenditure relatively stable [60], in accordance with the concept of “net zero-sum” [63].

Notably, our 40 Hz tACS results were highly concordant with our recent work of 5 Hz analog [43]. We thus conclude that narrow-band tACS in fact interferes with underlying neural synchronization across broad spectra (spectrum-unspecific), which in turn augments contralateral oscillatory powers. The neural spectral impact of tACS diminishes as the separation from the default frequency (i.e., 40 Hz in this research) increases. Since entrainment theory is based on electrophysiological research via invasive recording, how to reconcile the two seemingly contradictory observations? In fact, the “entrainment” may not be reflected in the magnitude of power, but in the connectivity strength at the delivered frequency band (spectrum-specific) (see below).

### 4.2. Connectivity changes associated with tACS treatment

After 40 Hz tACS treatment, the lagged phase synchronization at the gamma range significantly increased between the frontal and parietal regions. The increase in lagged coherence did not pass the statistical challenge. The pattern replicated our recent work of 5 Hz tACS [42]. Compelling evidence indicates that tACS can modulate the timing of neuronal spiking activity [32],[33],[35]. When tACS is applied to two distinct brain regions, it is anticipated to enhance synchronization of neural firing in these areas, with the timing guided by the applied alternating currents and the corresponding oscillatory frequencies. Mathematically speaking, the formula for phase synchronization closely resembles that of coherence, with the sole distinction being the inclusion of a normalization procedure aimed at mitigating the impact of power. Interestingly, similar to our previous work [42], the index based on lagged phase synchronization proved to be more robust than that derived from lagged coherence, indicating that what was “entrained” was not power but phase relationship.

In addition, since the recorded EEGs spanned 11 sessions of tACS treatment, not real-time, the significant inter-regional interactions suggest the activation of a plasticity mechanism. Similarly, Vossen and colleagues investigated the after-effects of alpha tACS and claimed that tACS's modulatory influence was driven by plasticity as opposed to entrainment [64]. To conclude, at the large-scale network level, the inter-regional effects of tACS were mediated by phase synchronization (crosstalk) and subsequent involvement of neural plasticity, not by the concurrent entrainment of powers. Our separate tACS studies altogether offer novel insights to clarify the debates surrounding the applicability of entrainment theory. In the context of the observed power desynchronization, this increase in connectivity may reflect a potential neural mechanism through which gamma-range tACS supports improved neuropsychological functions. This interpretation is consistent with the established role of gamma-band synchronization within the fronto-parietal network in supporting a wide range of cognitive processes, including attention and working memory [8],[65],[66].

Previous studies investigating the effects of tACS on functional connectivity indices have shown inconsistent patterns, such as the direction of connectivity change and spectral specificity. For example, frontal theta tACS has been reported to enhance phase-locking values between frontal and posterior regions across a broad spectrum [67], whereas delta tACS increased phase lag indices in the theta range rather than in delta [68]. Bilateral gamma tACS was found to decrease inter-hemispheric connectivity, modulated by different phase lags [69]. Other studies have shown spectral specificity in the modulation of functional connectivity, indicating that different frequencies can selectively enhance connectivity across various brain networks [70]. Comparative research has raised concerns about the validity of using scalp signals to study cortical connectivity, as cortical sources typically do not project radially to the scalp, which can lead to inaccurate or incomplete representations of brain network interactions [71],[72]. The striking consistency observed in our separate tACS studies underscores the advantages of a brain-based tomography approach compared to scalp-based, topographical methods.

The correlation analyses revealed that changes in functional connectivity within the frontoparietal network were nearly significantly associated with improvements in Letter Fluency (r = 0.33, p = 0.056). This finding is consistent with previous evidence linking linguistic material processing to the functioning of the left-lateralized frontoparietal network [73]. In contrast, Design Fluency and Trail B primarily tap spatial processing, which is right-hemisphere dominant, explaining their lack of significant correlations with connectivity changes [74]. Improvements in Trail A, which primarily reflects sensorimotor speed, were also unrelated to connectivity changes, as expected. The observed gains in Design Fluency, Trail A, and Trail B may largely reflect practice effects rather than network-specific modulation.

We acknowledge several limitations in this preliminary report, including the potential influence of practice effects on neuropsychological test performance and the absence of a sham control group to separate genuine treatment effects from placebo responses. However, implementing a valid sham control in tACS research remains challenging because sham and active stimulation often produce distinguishable skin sensations, making blinding difficult to maintain, particularly in repeated-session paradigms. In addition, practice effects are unlikely to selectively alter contra-hemispheric oscillatory power or functional connectivity within narrow spectral bands, which reduces the likelihood that the observed spectrum-specific changes can be fully explained by repeated testing alone. Importantly, regarding functional connectivity, our previous 5 Hz tACS study showed changes confined to the stimulated frequency band, and the current 40 Hz protocol similarly induced alterations restricted to the gamma range. Taken together, these observations support a stimulation-specific mechanism rather than a task learning effect. Although the 10–20 EEG system has a limited number of electrodes, nonetheless, given that both EEG and tACS are low-resolution tools (the latter is evident from simulation computation) [75],[76], increasing the electrode count may not yield significant advantages. Given the positive outcomes and the importance of clarifying the neural mechanisms underlying frontoparietal tACS, sham- and healthy-controlled clinical trials are warranted to further validate the conclusions of this preliminary EEG report.

## 5. Conclusions

This study investigated the neural effects of 40 HZ tACS at 2.0 mA over the left frontoparietal network (F3 and P3) by comparing EEG recordings before and after a 11-session treatment. To realize the tomography approach, the EEG registered from the scalp was converted to brain signals using eLORETA. The resulting neural changes were consistent with our previous report of 5 Hz tACS over the right hemisphere. In summary, the neural patterns subsequent to tACS included regional desynchronization across broad spectra, accompanied by contralateral enhancement of power, and an enhancement of inter-regional phase synchronization at the default frequency. We thus infer that the “entrainment” by tACS may exert its influence on the functional connectivity, not oscillatory power.

## Use of AI tools declaration

The authors declare they have not used Artificial Intelligence (AI) tools in the creation of this article.
